# The relationship between polycystic ovary syndrome and gynecological cancers: neurotransmitter metabolism changes and immune regulation

**DOI:** 10.3389/fimmu.2025.1578470

**Published:** 2025-06-04

**Authors:** Dongning Wu, Yue Zhang, Congying Wu, Bo An, Xin Wang, Jinxia Ni, Min Chen

**Affiliations:** ^1^ Faculty of Chinese Medicine and State Key Laboratory of Quality Research in Chinese Medicines, Macau University of Science and Technology, Macau, China; ^2^ Dongzhimen Hospital, Beijing University of Chinese Medicine, Beijing, China; ^3^ Xidan Clinic of Guang’anmen Hospital, Chinese Academy of Chinese Medicine Science, Beijing, China; ^4^ Guang’anmen Hospital, Chinese Academy of Chinese Medicine Science, Beijing, China; ^5^ The First Affiliated Hospital of Guangzhou University of Chinese Medicine, Guangzhou, China; ^6^ Guangdong Clinical Research Academy of Chinese Medicine, Guangzhou, China; ^7^ Data Center of Chinese Medical, China Academy of Chinese Medical Sciences, Beijing, China

**Keywords:** polycystic ovary syndrome, tumors, immune cells, neurotransmitters, ovarian cancer

## Abstract

Polycystic ovary syndrome (PCOS) is a common endocrine disorder affecting approximately 10% of middle-aged women worldwide. It is characterized by hirsutism, anovulation, and polycystic ovaries. Various factors, including environmental toxins, inflammation, oxidative stress, and insulin resistance, contribute to the progression of this condition. PCOS is also associated with metabolic disturbances, such as abnormal hormone and neurotransmitter metabolism, leading to obesity, hyperandrogenemia, and type 2 diabetes. Among female cancers, breast cancer, endometrial cancer, and ovarian cancer have high incidence rates and pose significant threats to women’s health. Studies suggest a potential link between PCOS and these gynecological cancers. Consequently, hormonal alterations in PCOS patients may influence tumorigenesis and metastasis. Moreover, PCOS is characterized by chronic low-grade inflammation, including dysregulated pro-inflammatory cytokine secretion, increased immune cell proliferation, and endothelial dysfunction. These factors contribute to cancer development, primarily through impaired immune metabolism, preventing effective tumor cell clearance and facilitating metastasis. This review synthesizes current knowledge on the mechanistic links between PCOS and gynecological cancers, focusing on the roles of immune cell dysfunction, aberrant cytokine secretion, and neurotransmitter metabolism. Therapeutic strategies, including hormonal interventions, insulin sensitizers, and lifestyle modifications, may mitigate cancer risk by modulating these pathways. This review highlights critical gaps in understanding PCOS-related oncogenesis and advocates for further research to elucidate molecular mechanisms and optimize clinical management.

## 1 Introduction

Polycystic ovary syndrome (PCOS) is a polygenic endocrine disorder influenced by genetic, hormonal, and environmental factors. It affects up to 10% of women globally, significantly impacting reproductive health, particularly infertility, acne, and psychological disorders (1). The diagnosis of PCOS follows the 2003 Rotterdam ESHRE/ASRM criteria, including oligo/anovulation, hyperandrogenism, and ovarian morphology with at least 20 follicles on ultrasound (2). PCOS is a multifactorial condition associated with intrauterine growth abnormalities, obesity, and metabolic syndrome (3, 4). PCOS patients exhibit significantly elevated levels of CRP and IL-18, contributing to a chronic inflammatory state and increasing cancer risk (5). Persistent inflammation accelerates adipocyte necrosis, triggering further immune cell infiltration and cytokine release, perpetuating a vicious cycle. Dysregulated immune activation and abnormal immune responses can also disrupt antibody and complement levels, leading to immune dysfunction. When immune surveillance fails, cancer cells can proliferate and metastasize.

Animal studies demonstrate that hyperhomocysteinemia-induced macrophage polarization toward the M2 phenotype promotes adipose tissue inflammation in PCOS mice, consistent with previous findings (6). PCOS patients show a shift from anti-inflammatory M2 macrophages to pro-inflammatory M1 macrophages, sustaining chronic inflammation (7). Additionally, dendritic cell proportions and immune factor expression decline in PCOS patients due to ovarian immune microenvironment disruption (8). Natural killer (NK) cells, crucial in innate immunity, may contribute to PCOS-related infertility. Clinical studies indicate reduced endometrial NK cell proportions in infertile PCOS patients (9). Moreover, T lymphocytes play a role in PCOS-related infertility, with Th1/Th2 imbalance potentially impairing oocyte quality and ovulation, increasing infertility and miscarriage risks. Recent studies utilizing the inverse variance-weighted model have revealed that PCOS is associated with five immune phenotypes, including B cells, NK cells, and monocytic myeloid-derived suppressor cells (M-MDSCs) (10).

Despite increased research efforts and funding, the precise mechanisms underlying PCOS remain controversial (11). Its etiology involves complex genetic and environmental factors, with numerous implicated genes suggesting a hereditary component (12). Environmental factors, including smoking, poor diet, and sedentary lifestyles, also contribute to PCOS. Obesity is a particularly significant concern, with studies indicating that obese women have a higher PCOS risk and more severe complications (13). Some researchers propose a genetic basis for PCOS, as first-degree relatives of PCOS patients often exhibit hyperandrogenemia and type 2 diabetes, with certain familial genes significantly correlating with PCOS risk (14).

Given the heterogeneity of PCOS and its endocrine-metabolic abnormalities, clinical outcomes and cancer risks may vary. Studies indicate that PCOS patients have an elevated risk of endometrial cancer, underscoring the need for preventive measures and early detection (15). The link between PCOS and gynecological malignancies may involve human papillomavirus (HPV) infection, endocrine dysregulation, and infertility-related factors. PCOS-related infertility is another potential cancer risk factor, as increased age at first pregnancy correlates with higher cancer risk (16). Additionally, PCOS-related obesity may further contribute to cancer risk (17). Some hypotheses suggest overlapping genetic abnormalities between PCOS and cancer, involving hydroxysteroid dehydrogenases, platelet-derived growth factor receptors, and high-mobility group protein 2 (18).

The adverse consequences of PCOS may stem from dysregulated hormone metabolism, particularly insulin resistance, leading to obesity and exacerbating PCOS symptoms (19). Obesity-induced fat accumulation and potential binge eating tendencies in PCOS patients further contribute to weight gain, psychological distress, depression, and cognitive dysfunction (20). Current clinical management focuses on weight control and psychological well-being to mitigate PCOS symptoms and reduce mental health risks (21). Emerging treatment approaches emphasize vitamin and mineral supplementation, acupuncture, and yoga, which offer promising therapeutic effects with minimal adverse risks, making them increasingly popular among PCOS patients (22). This review aims to elucidate the pathophysiological mechanisms linking PCOS to gynecological cancers, focusing on neurotransmitter metabolism and immune cell involvement.

## 2 Pathogenesis of PCOS

Despite extensive fundamental research efforts, the precise pathogenesis of PCOS remains unclear. Several theories have been proposed to explain the development of PCOS, with hyperandrogenemia, insulin resistance, and obesity being recognized as key contributing factors. However, the exact triggers of these mechanisms remain a subject of debate, with scientists suggesting that genetic and environmental factors play a significant role (23). A hallmark feature of PCOS is obesity, leading researchers to hypothesize that excessive adipose tissue accumulation is a crucial driver of PCOS development. Increased visceral fat deposition elevates circulating free fatty acid levels, contributing to hyperglycemia and subsequent hyperinsulinemia, which stimulates luteinizing hormone receptor activity (24). Leptin, a peptide hormone involved in energy metabolism, may also be implicated in PCOS pathogenesis. Leptin suppresses the expression of aromatase mRNA in granulosa cells, inhibiting the conversion of androgens to estrogens and resulting in androgen accumulation (25). In addition, adiponectin is also an important metabolic mediator. Under the unstable secretory environment of adipocytes, the increased release of leptin and decreased secretion of adiponectin act synergistically, exerting profound effects on the mass distribution and function of adipose tissue (26).

Insulin resistance is an independent pathogenic factor in PCOS, as insulin modulates hormone production, particularly androgens, and influences ovarian polycystic morphology and P450c17 enzyme activation. These factors collectively lead to increased androgen concentrations. Furthermore, insulin reduces sex hormone-binding globulin levels, leading to testosterone accumulation in the ovaries, while also promoting lipogenesis and inhibiting lipolysis, thereby facilitating adipose tissue deposition (27). Hyperandrogenism, insulin resistance, and estrogen imbalance are endocrine disorders that may influence the tumor microenvironment through multiple mechanisms. First, elevated androgen levels can directly promote tumor cell proliferation and survival. Second, hyperinsulinemia resulting from insulin resistance may enhance tumor cell growth and survival by activating key signaling pathways such as PI3K/AKT (28). Additionally, excessive estrogen stimulation can further support tumor growth and metastasis by promoting angiogenesis and regulating inflammatory cytokines. These endocrine abnormalities may also alter the immune cell function, modifying the immune landscape of the tumor microenvironment, thereby influencing tumor initiation and progression (29).

Recent studies have identified a critical role for immune cells in the onset and progression of PCOS, particularly in the context of chronic low-grade inflammation. PCOS patients exhibit elevated levels of cytokines such as IFN-γ, IL-6, and IL-18 (30). Immune cell dysregulation may also contribute to PCOS-related infertility. T lymphocytes regulate ovarian immune responses and cytokine release; however, hormonal imbalances in PCOS disrupt peripheral blood and ovarian T cell subpopulations (31). Additionally, B lymphocytes have been implicated in PCOS pathogenesis, not only in antibody secretion but also in antigen presentation regulation. In the context of obesity and glucose intolerance-associated insulin resistance, B cells play a crucial role by activating T cells and secreting pathological antibodies that exacerbate insulin resistance (32). In one study, infusion of CD19 antibodies into PCOS mice significantly reduced peripheral B lymphocyte counts, decreased cystic follicle numbers, and increased corpus luteum formation, suggesting that these immune cells, along with antibodies and cytokines, could serve as potential therapeutic targets for PCOS (7). Na Aru et al. conducted a Mendelian randomization study assessing associations between PCOS and 731 immune cell subtypes, identifying four immune phenotypes significantly correlated with PCOS risk, particularly memory B cells, providing robust causal evidence for clinical applications (33). In recent years, studies have revealed that patients with PCOS may also have multiple mechanisms that promote tumor immune escape, including T cell dysfunction, increased M2 macrophages, and activation of the PD-1/PD-L1 immune checkpoint (34). Specifically, the common endocrine and metabolic abnormalities in PCOS patients can lead to T cell exhaustion and dysfunction, weakening their anti-tumor immune response. Meanwhile, the accumulation of M2 macrophages in the tumor microenvironment secretes various immunosuppressive factors, further supporting tumor growth and metastasis. Additionally, the activation of the PD-1/PD-L1 pathway inhibits T cell activation, allowing tumor cells to evade immune surveillance (35). The functions of these immune cells are listed in **Table 1**.

**Table 1:** The role of immune cells in PCOS patients.

Immune cell	Cytokines	Specific function	References
CD11c+ HLA- DR+ DCs	Th17, Th1	Superior follicle selection	(36)
Th1 cells	IFN-γ, IL-2	Ovulation	(2)
Th2 cells	IL-4, IL-10	Ovulation	(2)
Treg cells	IL-10, TGF-β	Proinflammatory reaction	(37)
Neutrophil	IL-1	Inflammatory response	(23)
B lymphocyte	CCL-22, IL-2	Immune response	(23)
Macrophage	IL-1, TNF-α	Inflammatory response	(38)
Natural killer cells	IFN-γ, TNF-α	Kill pathogens	(38)
Dendritic cells	ICAM-1, CD40	Antigen presentation	(38)

**Figure 1:**
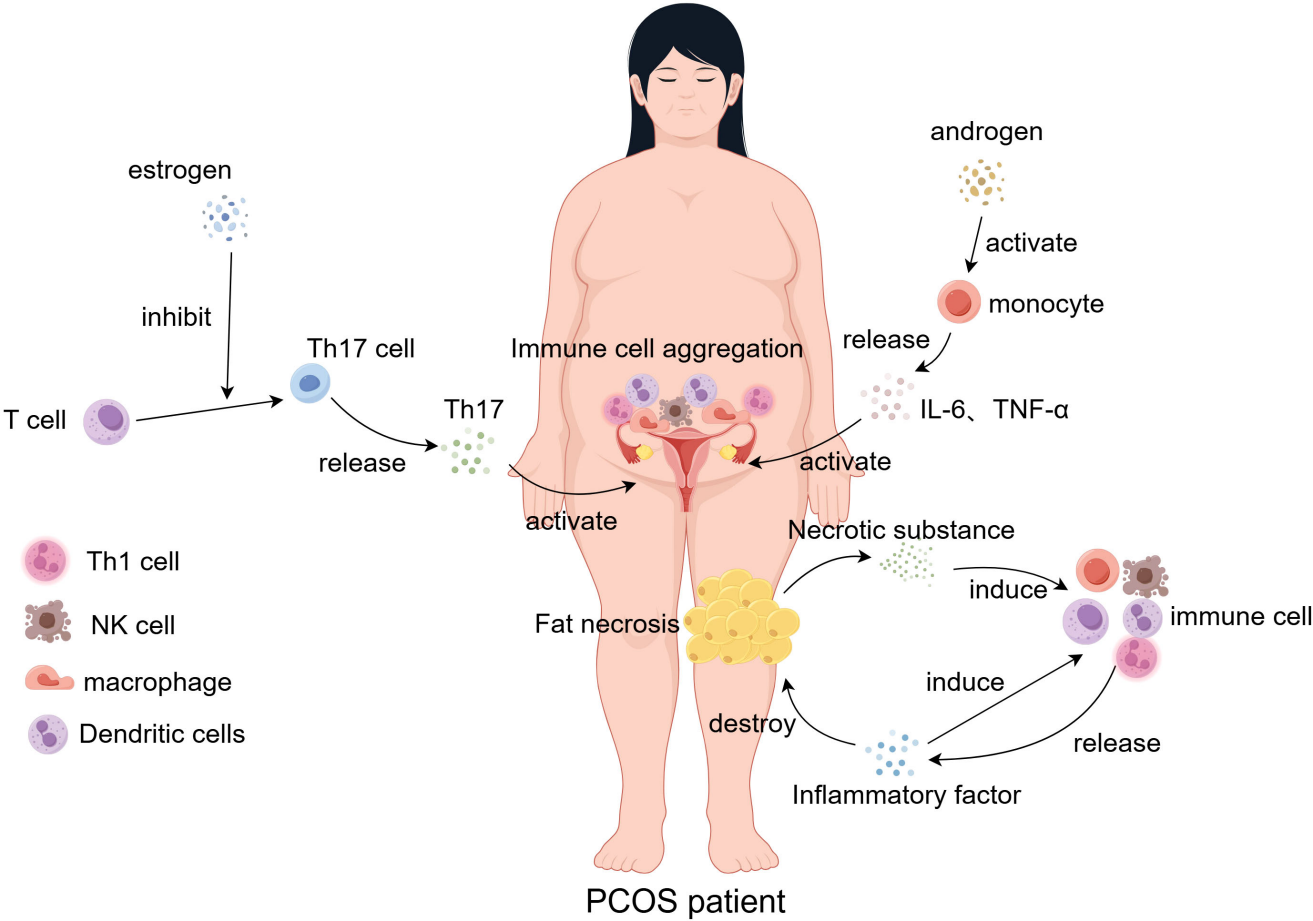
In PCOS patients, the inflammatory response of immune cells within the endometrium and adipose tissue is dysregulated under the influence of estrogen and androgen stimulation. Concurrently, immune cell-secreted cytokine levels are disrupted. Necrosis of adipose tissue results in the aggregation of immune cells, which release cytokines and chemokines that further recruit additional immune cells to the necrotic adipose tissue, exacerbating inflammation and perpetuating adipose tissue damage.

The classical pathogenic mechanisms of PCOS have been widely recognized; however, emerging research suggests that disrupted enzymatic activity during ovulation may also contribute to its development. Specifically, the luteinizing hormone (LH) surge activates members of the matrix metalloproteinase family (MMP2, 9, 14, 19), including ADAMTS-1, which plays a crucial role in ovulation. Before ovulation, ADAMTS-1 levels increase, facilitating extracellular matrix degradation and follicular rupture. However, genetic abnormalities affecting ADAMTS-1 expression may impair follicular rupture, leading to anovulation and subsequent follicular cyst formation (39) (**Figure 1**).

PCOS is not only an endocrine disorder but also associated with genetic abnormalities (such as PTEN, KRAS, FOXO1) that may contribute to tumorigenesis. Specifically, PTEN, a tumor suppressor gene, when lost or mutated, leads to the persistent activation of the PI3K/AKT signaling pathway, promoting cell survival and proliferation. Meanwhile, the aberrant activation of KRAS drives malignant transformation through the MAPK cascade (40). FOXO1, an important transcription factor that regulates the cell cycle and apoptosis, is often associated with cell regulatory imbalances when its expression is abnormal. In PCOS patients, chronic hyperinsulinemia and hormonal imbalances may further exacerbate these molecular abnormalities, enhancing the activation of the PI3K/AKT and MAPK pathways, thus increasing the risk of cancer development. These findings suggest that a deeper exploration of the interactions between PCOS-related genes and key signaling pathways is crucial for understanding its carcinogenic mechanisms and developing targeted therapeutic strategies.

### 3.1 POCS and breast cancer

Epidemiological studies suggest that prolonged exposure of breast epithelial tissue to estrogen is associated with an increased risk of breast cancer, with factors such as menarche timing and menopause being significant risk determinants (41). Estrogen plays a regulatory role in immune cells, particularly macrophages, facilitating their polarization into distinct phenotypes depending on the microenvironment (42). Additionally, estrogen stimulates IL-6 secretion and drives T lymphocyte differentiation into Th17 cells, exacerbating systemic inflammatory responses (38). PCOS is closely associated with chronic inflammation, characterized by elevated levels of pro-inflammatory cytokines such as IL-6 and TNF-α. These cytokines not only promote systemic inflammation but also play a significant role in the tumor microenvironment (TME), potentially influencing cancer development and progression in PCOS patients. IL-6 is a pleiotropic cytokine involved in immune responses, inflammatory reactions, hematopoiesis, and other key physiological processes. It plays a crucial role in chronic inflammation, autoimmune diseases, and cancer. TNF-α is another pro-inflammatory cytokine that can induce the expression of PD-L1, thereby leading to an immunosuppressive state within the TME. Studies have shown that TNF-α can upregulate PD-L1 expression in various cell types, including cancer cells and mesenchymal stem cells, thus inhibiting the proliferation and activation of CD8+ T cells. The interaction between IL-6, TNF-α, and PD-L1 forms an immunosuppressive TME, allowing tumors to evade immune surveillance and promoting their progression. A deeper understanding of these mechanisms could help identify potential therapeutic targets, such as cytokine signaling pathways and immune checkpoint molecules, providing new treatment strategies for managing cancer risk in PCOS patients (36).

Animal studies have demonstrated a potential link between endocrine-metabolic disorders in PCOS and benign breast disease or breast cancer. In one study, 75% of PCOS rats and 33.33% of control group rats developed benign mammary tumors with well-defined and highly mobile masses. Histological examination revealed proliferative mammary lesions, indicating a significantly higher prevalence of benign breast tumors in postmenopausal PCOS rat models, likely due to hormonal imbalances (43). However, conflicting perspectives exist regarding the association between PCOS and breast cancer. Some studies suggest no increased cancer risk in PCOS patients. Chittenden’s review found no significant correlation between PCOS and cancer incidence, and Shobeiri’s study of 45,470 participants concluded that PCOS does not elevate cancer risk (44). A 2015 Danish cohort study involving 12,070 PCOS patients also found no significant difference in breast cancer risk compared to the general population (45).

Moreover, PCOS patients often undergo oral contraceptive (OC) therapy to regulate menstrual irregularities and hyperandrogenaemia, potentially increasing breast cancer risk. One study analyzing 54 individuals found a slight elevation in cancer risk with recent or ongoing OC use, with long-term use significantly exacerbating this risk. Hunter’s research on 110,000 participants similarly reported a mild increase in breast cancer risk, correlating with age, obesity, and smoking (46). Despite these risks, OCs remain a primary treatment for PCOS, particularly for menstrual dysfunction and hyperandrogenaemia. Furthermore, elevated androgen, insulin, and insulin-like growth factor (IGF) levels in obese PCOS patients may contribute to breast cancer progression. Androgens can directly stimulate AR-positive cancer cells, while insulin and IGF promote mitotic activity in cancer cells (47).

### 3.2 PCOS and endometrial cancer

Endometrial remodeling is regulated by sex hormones and plays a critical role in reproductive health. Immune cells and cytokines, including CD56^+^ uterine natural killer cells and cytotoxic T cells, influence endometrial function through the secretion of IL-15 and IL-10 (48). However, in PCOS patients, both the proliferative and secretory phases of the endometrium are affected by prolonged estrogenic exposure, while progesterone action during the secretory phase is diminished. This hormonal imbalance leads to endometrial damage, hyperplasia, and inflammation, increasing the risk of malignant transformation (49).

Endometrial cancer is a leading cause of cancer-related mortality in middle-aged women, characterized by high malignancy and rapid progression. The first report on the association between PCOS and endometrial cancer was published in 1949, and since then, this link has become a focus of scientific research. Endometrial cancer is categorized into two types, with Type I endometrioid adenocarcinoma comprising 80% of cases and closely associated with estrogenic levels (50). Additional risk factors include menarche timing, menopause, anovulation, hypertension, and diabetes (51). Insulin and IGF have been identified as proliferative agents in the endometrium, contributing to its thickening and playing a role in endometrial carcinogenesis (52).

A study examining endometrial tissue from endometrial cancer patients found increased insulin levels and IGF receptor and protein expression in epithelial and stromal cells, suggesting a role in cancer cell proliferation and differentiation (53). Dockerty’s retrospective study of 43 PCOS patients identified 16 cases of endometrial cancer, with the majority occurring in women under 40 years old. Another retrospective study of 2,573 infertile women found that 24 exhibited endometrial abnormalities, including 20 cases of endometrial hyperplasia and 4 cases of endometrial cancer, highlighting a potential increased cancer risk in infertile PCOS patients (54).

Despite limited large-scale studies, Ramzy’s histological biopsy of 15 ovaries from patients diagnosed with endometrial cancer before age 40 revealed an 11.1% prevalence of endometrial carcinoma linked to PCOS (55). The incidence of endometrial cancer in young women remains low; however, a survey identified risk factors among endometrial cancer patients under 40 years old, including obesity (17.9%), hypertension (42.1%), diabetes (21.1%), and PCOS (31.2%), emphasizing the strong correlation between PCOS and endometrial cancer (56). A long-term retrospective study in the UK analyzing 319 PCOS patients and 1,060 controls reported a 5.3% endometrial cancer incidence rate among PCOS patients (57). It is widely accepted that chronic anovulation and prolonged estrogen exposure in PCOS patients promote endometrial cancer progression (58).

**Figure 2:**
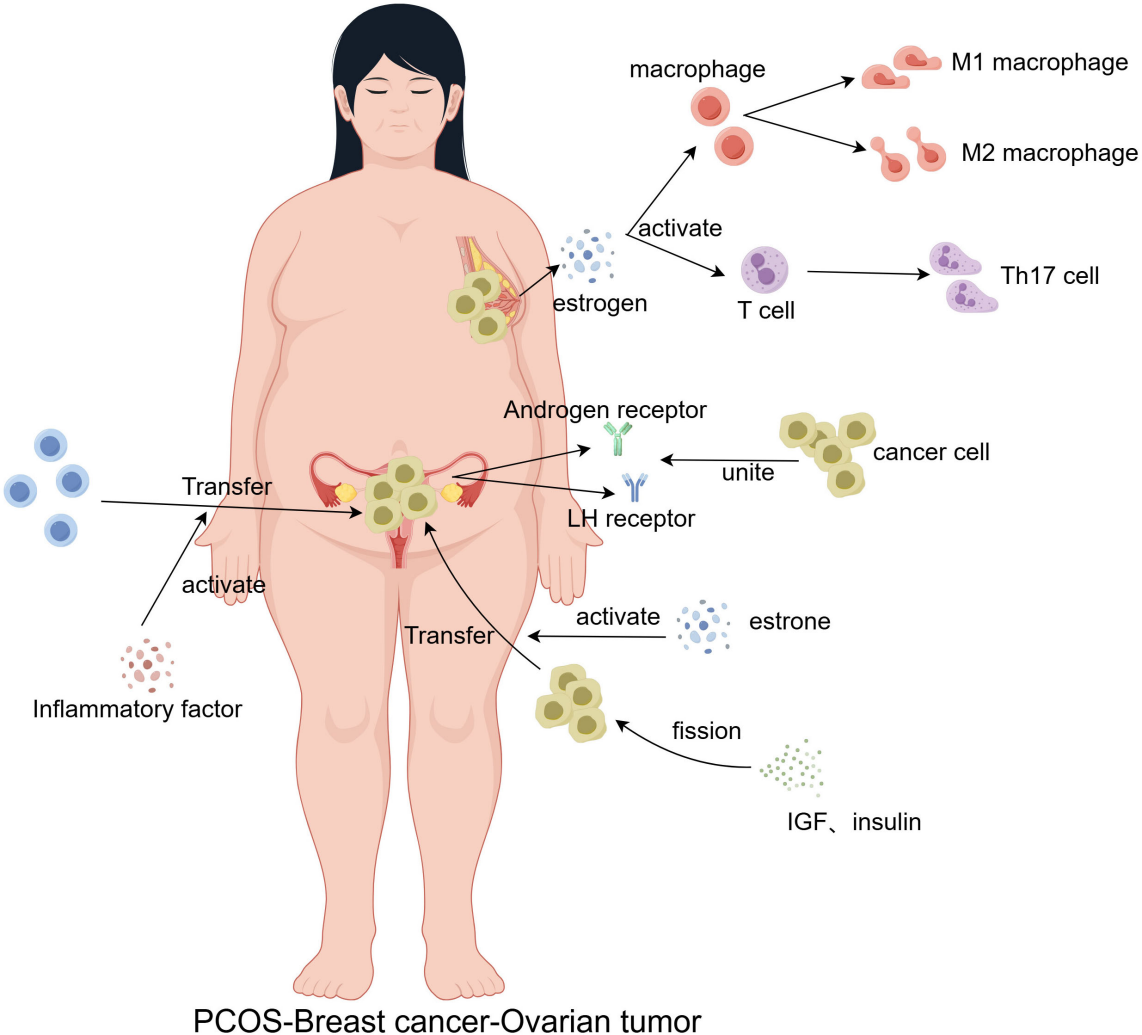
Chronic systemic inflammation in PCOS patients serves as a persistent stimulus that contributes to the malignant transformation of normal cells. Additionally, disruptions in estrone and estrogen levels promote immune cell polarization, as well as the mitosis, proliferation, differentiation, and metastasis of cancer cells. Increased expression of luteinizing hormone (LH) and androgen receptors further enhances cancer cell binding, facilitating tumor metastasis.

Compared to healthy women, PCOS patients exhibit increased androgen receptor expression in the endometrium, facilitating cancer cell implantation and metastasis. Additionally, elevated luteinizing hormone (LH) receptor expression in the endometrium of PCOS patients with endometrial cancer further supports cancer cell colonization. A study evaluating endometrial cancer risk in Thai PCOS patients found that when endometrial thickness exceeded 7 mm, the cancer risk increased to 8.7% (59). Moreover, obesity, insulin resistance, and diabetes further contribute to endometrial cancer risk, particularly in postmenopausal obese PCOS patients, where increased adipose tissue aromatization elevates circulating estrone levels, enhancing tumor metastasis potential (60) (**Figure 2**).

### 3.3 PCOS and ovarian cancer

In ovarian tissue, the stromal compartment is primarily composed of macrophages, neutrophils, and B lymphocytes, along with some lymphatic and blood vessels. Immune cells within the ovary perform multiple functions, including antigen presentation, protease release, and cytokine secretion (61). Studies have shown that monocytes infiltrating the ovary can trigger immune responses, stimulating androgen production in patients with PCOS. The prolonged impact of these factors on the body may lead to cellular malignant transformation, ultimately contributing to ovarian cancer development.

Ovarian cancer is one of the most common malignancies among women, with approximately 150,000 deaths reported annually. It is well established that ovarian cancer development is closely associated with hormonal changes in the body. Moreover, a reduced or irregular menstrual cycle significantly increases carcinogenic risk (62). In ovarian epithelial cells of patients with ovarian cancer, follicle-stimulating hormone (FSH), luteinizing hormone (LH), and androgen receptors are markedly elevated (63). Studies indicate that ovarian tissues from women with menstrual disorders exhibit DNA hypomethylation and produce miRNAs identical to those found in ovarian cancer, suggesting genetic mutations (64).

Furthermore, genetic abnormalities have been detected in various bodily fluids and tissues of PCOS patients, indicating a significantly increased cancer risk (65). Some studies propose that ovarian cancer occurrence may be linked to lifestyle factors such as obesity in PCOS patients, with poorer lifestyle habits correlating with a higher cancer risk. However, conflicting findings exist. Some researchers argue that there is no significant association between PCOS and ovarian cancer risk. A cohort study reported that PCOS patients with menstrual irregularities and prolonged menstrual periods did not exhibit an increased risk of ovarian cancer (66).

Conversely, Yin et al. suggested that Swedish PCOS patients had a higher risk of cancer, though they acknowledged that increasing the number of participants in the cohort study would enhance the reliability of their findings (67). An Australian cohort study analyzed risk factors associated with hyperandrogenism and found no evidence linking PCOS or acne to ovarian cancer. However, a positive correlation was observed between PCOS and serous borderline tumors, and the use of testosterone supplements increased the risk of ovarian cancer in PCOS patients (66). Schildraut et al. evaluated 476 ovarian cancer patients aged 20–54 years and found that women with PCOS had a 2.5-fold increased risk of developing ovarian cancer. This risk was closely associated with oral contraceptive use and obesity. However, only 31 ovarian cancer patients had been diagnosed with PCOS before the study (68).

Recent cohort studies have shown a statistically significant association between PCOS and an increased risk of gynecological cancers, particularly endometrial cancer and ovarian cancer (69, 70). In these studies, PCOS patients exhibited a higher incidence of endometrial cancer, which is partly attributed to chronic anovulation, hyperinsulinemia, and obesity, factors commonly present in PCOS patients. Moreover, recent evidence suggests that different PCOS phenotypes may have distinct cancer risk profiles. For example, the classic phenotype characterized by hyperandrogenism and menstrual irregularities appears to be significantly associated with an elevated risk of endometrial cancer, while its association with ovarian cancer is more moderate, and the conclusions remain inconclusive. These findings highlight the importance of stratifying PCOS patients based on phenotype to improve risk assessment and develop more targeted monitoring and prevention strategies for this high-risk population.

### 3.4 PCOS and uterine sarcoma

The most common types of uterine sarcomas include leiomyosarcoma, endometrial stromal sarcoma (ESS), and carcinosarcoma. Among them, ESS is highly hormone sensitive. Studies have revealed that patients undergoing bilateral salpingo-oophorectomy have a significantly lower postoperative recurrence risk than those who retain their ovaries and fallopian tubes (71). In ESS, estrogen and progesterone receptor levels are markedly elevated. Furthermore, approximately 30% of patients with uterine carcinosarcoma exhibit increased levels of these hormone receptors, particularly in cases associated with PCOS (72). Although most cases of carcinosarcoma occur postmenopause, a subset of PCOS-related carcinosarcoma cases has been reported in patients younger than 40 years (73).

In recent years, although epidemiological data remain limited, preliminary studies suggest that patients with PCOS, due to prolonged estrogen overstimulation, may face an increased risk of uterine sarcoma. Some cohort studies and case-control analyses have found that endocrine disorders in PCOS patients, such as chronic anovulation and prolonged estrogen exposure, may promote the malignant transformation of uterine leiomyomas, thereby increasing the incidence of sarcomas. Meanwhile, experimental studies indicate that excessive estrogen stimulation may activate key signaling pathways, such as PI3K/AKT and MAPK, promoting abnormal cell proliferation and genomic instability, which in turn may drive the malignant transformation of uterine fibroids and lead to highly invasive uterine sarcomas (74). These findings provide new insights into the potential causal relationship between PCOS and uterine sarcoma and underscore the importance of early risk assessment and intervention for PCOS patients.

## 4 Neurotransmitter metabolism in PCOS

PCOS is one of the leading causes of infertility in reproductive-aged women. This condition is strongly associated with metabolic abnormalities in neurotransmitters such as γ-aminobutyric acid (GABA), glutamate, dopamine, and acetylcholine, which are involved in the neuroendocrine regulation of PCOS. Neurotransmitter dysregulation can have profound psychological effects, a common symptom in PCOS patients. Studies have shown that individuals with PCOS have a significantly higher prevalence of depression, as well as an increased risk of eating disorders and anxiety disorders compared to healthy individuals (75). Therefore, monitoring neurotransmitter levels in PCOS patients and modulating their expression may hold promise for improving PCOS symptoms.

### 4.1 Gonadotropin-releasing hormone

Women with PCOS often experience infertility, and these patients exhibit elevated luteinizing hormone (LH) levels, indicating increased secretion of GnRH and anti-Müllerian hormone (AMH). Excessive intrauterine AMH may adversely affect fetal development. A study revealed that compared to women without reproductive disorders, pregnant women with PCOS exhibit elevated AMH concentrations. Similarly, animal studies have shown that high AMH levels lead to maternal abnormalities, resulting in neuroendocrine phenotypes resembling PCOS in adult offspring. Treatment with GnRH antagonists was found to reverse these symptoms (76). The elevation of GnRH in PCOS patients is primarily due to reduced sensitivity to negative feedback from sex hormones, leading to increased LH and androgen levels, which form a pathological cycle that exacerbates PCOS symptoms. Disrupting this cycle can effectively alleviate PCOS symptoms (77).

In an animal study, activation of GnRH neurons using designer receptors exclusively activated by designer drugs (DREADDs) resulted in neuroendocrine disturbances, including pathological hormone secretion and disrupted estrous cycles resembling PCOS. Increased numbers of preantral follicles were observed, indicating impaired ovulatory activity. This study confirmed that hyperactivity of hypothalamic GnRH neurons is a key factor in hormone dysregulation and ovarian dysfunction in PCOS (78).

Kisspeptin neurons are considered the pulse generator of GnRH, inducing pulsatile secretion via autocrine or paracrine mechanisms (79). Studies have shown that blocking neurokinin B stimulation alleviates GnRH pulsatility, leading to reductions in LH and androgen levels. In a study on PCOS patients, administration of a neurokinin B inhibitor for one month significantly reduced LH and androgen levels compared to baseline (80). Kisspeptins, encoded by the Kiss1 gene, regulate the hypothalamic-pituitary-gonadal axis by binding to receptors on GnRH neurons. A human study measuring biochemical parameters in PCOS patients found significantly higher serum Kisspeptin and testosterone levels compared to controls, indicating a positive correlation between Kisspeptins and PCOS progression (81).

### 4.2 Acetylcholine

Interestingly, irritable bowel syndrome (IBS) is highly prevalent among PCOS patients. However, the underlying mechanisms affecting gastrointestinal motility in PCOS remain unclear. Wanger et al. established a PCOS rat model using dihydrotestosterone and analyzed acetylcholine and carbachol expression levels in gastrointestinal tissues after 17 weeks. Results showed that acetylcholine responsiveness and contractile force in the gastric fundus and colonic muscle were significantly reduced in the PCOS group. A decrease in extracellular calcium ion responsiveness and MLC20 phosphorylation was also observed, indicating that PCOS may induce gastroparesis and reduce gastrointestinal contractility, primarily via diminished acetylcholine responsiveness (82).

In another study, PCOS rats treated with the traditional Chinese medicine Wushenhua formula for three estrous cycles exhibited significant metabolic changes. Serum metabolomics revealed reductions in creatine, creatinine, and glycerophosphoethanolamine, whereas lysine, ornithine, and acetylcholine levels increased in the hyperinsulinemic PCOS group. These findings suggest that the Wushenhua formula may alleviate PCOS symptoms by reducing inflammation and oxidative stress (83).

Endocrine dysfunction in PCOS is also implicated in cognitive impairment. The Notch signaling pathway has been suggested to regulate ovarian function and contribute to neurodegenerative diseases. Liraglutide, a drug with established neuroprotective effects, has been shown to improve cognitive dysfunction in PCOS patients. In a study, PCOS rats treated with liraglutide for 30 days exhibited hyperactivation of the Notch pathway and hippocampal neuronal degeneration, characterized by reduced acetylcholine levels and elevated serum insulin and testosterone levels. Liraglutide treatment suppressed Notch pathway activation and alleviated memory impairment, suggesting that aberrant hippocampal Notch signaling contributes to PCOS-associated cognitive dysfunction, which may be reversed by liraglutide (84).

Depression is also common in PCOS patients, potentially linked to histone deacetylation and DNA methylation, particularly in major depressive disorder. John et al. found that administration of letrozole to rats for 21 days induced hyperandrogenemia, ovarian cysts, and follicular atresia, accompanied by depressive-like behaviors. Increased DNA methyltransferase expression was observed in the hippocampus, along with elevated malondialdehyde and acetylcholine levels, indicative of neurodegeneration (85). These findings suggest that neuroinflammation and increased acetylcholine expression are hallmarks of depression associated with PCOS.

### 4.3 Dopamine

Dopamine is a neurotransmitter predominantly found in the brain, exerting its effects by binding to specific G protein-coupled receptors. Due to its instability, dopamine undergoes auto-oxidation, which may contribute to functional impairments in PCOS patients (37). A study comparing PCOS patients with healthy individuals measured glucose, LH, dopamine, and prolactin levels. PCOS patients exhibited significantly higher body mass indices, prolactin, dopamine, and LH levels, all of which positively correlated with PCOS severity (86). These findings suggest that elevated dopamine plays a role in PCOS pathogenesis.

Dopamine receptor 2 (DRD2) modulates dopamine-mediated inhibition of GnRH neuron excitability and prolactin secretion. Polymorphisms in DRD2 may contribute to PCOS progression. A genetic study analyzed 22 DRD2 variants in 212 Italian families and identified five novel variants (e.g., rs6277, rs4936274) significantly associated with PCOS risk, though further functional studies are required for confirmation (11).

Furthermore, measuring the dopamine metabolite homovanillic acid (HVA) can provide insights into dopamine metabolism in PCOS. Segos et al. analyzed urinary HVA levels in 35 PCOS patients and found significantly increased HVA excretion compared to healthy controls. A negative correlation was observed between plasma LH levels and HVA levels in healthy women (87), suggesting that dopamine’s inhibitory effect on LH secretion may be impaired in PCOS patients.

## 5 Discussion

PCOS remains a complex disorder, and its pathogenesis is yet to be fully understood by clinicians and scientists. Current research not only focuses on developing effective treatment strategies and pharmacological interventions for PCOS but also investigates its potential association with gynecological malignancies and the role of immune cells in PCOS-induced cancer. Given that cancer can severely impact both PCOS management and patients’ quality of life, it is crucial to explore this potential link further. Although the association between PCOS and cancer remains controversial due to limited research data, this should not deter clinicians from monitoring cancer incidence in PCOS patients. Most studies suggest a correlation between PCOS and the occurrence of gynecological tumors, while some clinical researchers argue that the connection is weak. This discrepancy highlights the need for further clinical and cohort studies involving larger populations to clarify the relationship. Recent findings indicate that PCOS is associated with dysregulation of immune cell function, abnormal cytokine levels, and chronic inflammation. Chronic inflammation, triggered by cytokine imbalances, immune dysfunction, and hormonal abnormalities, has been recognized as a significant factor in cellular transformation and tumorigenesis. Understanding the pathogenic mechanisms of immune cells in PCOS could provide insights into preventing and treating cancer-related complications in PCOS patients.

Currently, PCOS prevalence among reproductive-age women is increasing, and its lifelong complications pose significant challenges for both patients and the medical community. The genetic and pathophysiological mechanisms underlying PCOS require further in-depth investigation to develop more targeted treatment strategies (10). Notably, PCOS manifests with varying severity among individuals, complicating both diagnosis and treatment. Since its initial description by American physician Irvin F. Stein, PCOS has continuously evolved in its clinical presentation. Advances in modern medical technology and research have provided clinicians with a clearer understanding of the disease, making diagnosis and treatment more accessible (88). However, despite substantial technological progress, the exact pathogenesis of PCOS remains elusive, partly due to its heterogeneity, which complicates both diagnosis and classification (89). Given the diverse manifestations of PCOS, individualized treatment plans should be developed by multidisciplinary teams of medical specialists to optimize disease management and improve patients’ overall well-being.

In recent years, data from large cohort studies and laboratory research have consistently shown a close association between PCOS and the risk of gynecological cancers, particularly endometrial and ovarian cancer. PCOS patients often exhibit chronic anovulation, hyperandrogenemia, and insulin resistance, creating an endocrine and metabolic environment that leads to prolonged estrogen exposure of the endometrium, thereby increasing the risk of endometrial hyperplasia and malignant transformation. Epidemiological data indicate that the incidence of endometrial cancer is significantly higher in PCOS patients compared to non-PCOS populations. Regarding ovarian cancer, some studies suggest that PCOS may be associated with a mildly increased risk; however, the evidence remains controversial. Experimental studies indicate that abnormal activation of insulin and growth factor signaling pathways (such as PI3K/AKT) in PCOS may induce pro-tumorigenic signaling in ovarian cells, thereby promoting abnormal cell proliferation. Taken together, these latest epidemiological and experimental findings support the implementation of individualized cancer screening and prevention strategies for PCOS patients, particularly for different PCOS subtypes, to better manage their cancer risk.

For decades, pharmacological treatment of PCOS has been a focal point of research, and current clinical guidelines have established relatively comprehensive treatment protocols (90). Recent studies suggest that different types of hormonal interventions may significantly affect the cancer risk in PCOS patients. For example, oral contraceptives, by providing exogenous progestins, promote endometrial shedding, thereby effectively reducing the risk of endometrial hyperplasia and endometrial cancer induced by chronic anovulation; anti-androgen therapies, by lowering circulating androgen levels, may reduce androgen-mediated pro-carcinogenic signaling; and insulin sensitizers (such as metformin) help improve insulin resistance and lower hyperinsulinemia, both of which are believed to be closely associated with the development of certain types of cancer. Overall, these interventions exert protective effects by modulating the endocrine environment in PCOS patients, reducing chronic inflammation, and regulating cell proliferation, which may consequently lower the overall cancer risk. However, current evidence remains somewhat controversial, and the specific impact of various hormonal interventions on PCOS-related cancer risk has not been fully clarified (91). At the same time, psychological distress and infertility continue to present significant challenges in the management of PCOS. Although pharmacological interventions are commonly used to induce ovulation and improve fertility outcomes, their side effects and economic burden can cause considerable distress for patients. Consequently, alternative therapies such as acupuncture, moxibustion, and yoga have gained increasing attention in recent years. Some studies suggest that acupuncture not only effectively alleviates psychological stress in PCOS patients but may also enhance ovulatory function, offering a promising, non-invasive treatment option for infertile PCOS patients. These complementary therapies, due to their safety, convenience, and efficacy, are increasingly favored by PCOS patients seeking comprehensive management solutions.

In summary, while multiple treatment options are currently available for PCOS patients, further research is needed to clarify the specific impact of various interventions on PCOS-related risks. Additionally, personalized treatment strategies that incorporate alternative therapies may provide a more holistic management approach, potentially improving patients’ quality of life and long-term health. Further exploration of the PCOS treatment relationship with related malignancies is also required.
